# Associations Between Social Determinants of Health and Outcomes of Chronic Medical Conditions

**DOI:** 10.7759/cureus.67528

**Published:** 2024-08-22

**Authors:** Alice Hamilton, Alice A Beneke, Emily Meisel, Cristian Zhang, Hanzhi Gao, Jessica Portillo-Romero

**Affiliations:** 1 Internal Medicine, University of Florida College of Medicine, Gainesville, USA; 2 Internal Medicine, Albert Einstein College of Medicine, New York, USA; 3 Statistics, University of Florida, Gainesville, USA

**Keywords:** type 2 diabetes mellitus, essential hypertension, chronic disease management, food insecurity, social determinants of health

## Abstract

Social determinants of health, such as food insecurity, can significantly impact patient welfare, potentially increasing the prevalence of chronic illnesses while hindering their management, as shown in previous data collected by the National Health and Nutrition Examination Survey. This study aimed to investigate the association between food insecurity and other social determinants of health with hyperlipidemia, type 2 diabetes mellitus (T2DM), and hypertension. To that end, self-reported data on food security from clinical encounters and biological data from medical records were collected. This study utilized electronic medical record data from 349 patients aged between 18 and 85 years who answered two standard food insecurity screening questions. Each patient’s current diagnoses and lab values, including blood pressure, fasting low-density lipoprotein (LDL) cholesterol, and hemoglobin A1c (HbA1c), were then collected. Among patients facing food insecurity (n = 48), 55% were diagnosed with hypertension (p = 0.019), 45% with hyperlipidemia, and 27% with T2DM (p = 0.005). By comparison, these values for food-secure patients were 39%, 54%, and 13%, respectively (n = 301, p > 0.05). Regarding control of these chronic illnesses, hypertension (defined as blood pressure >135/85 mmHg per American Academy of Family Physicians (AAFP) guidelines) was observed in 12% of food-secure patients (n = 301, p > 0.05) and 42% of food-insecure patients (n = 48, p = 0.0204), whereas differences in control of hyperlipidemia and T2DM were insignificant. These results suggest that food-insecure patients are more likely to be diagnosed with hypertension and T2DM but are less likely than food-secure patients to be diagnosed with hyperlipidemia. Consistent with previous research, this study highlights the potentially increased health risks for patients experiencing food insecurity and calls for further efforts to screen patients for social determinants of health.

## Introduction

The United States Department of Agriculture (USDA) defines food insecurity as the “limited or uncertain availability of nutritionally adequate and safe foods or limited or uncertain ability to acquire acceptable foods in socially acceptable ways” [1]. According to a USDA report, 10.5% of U.S. households were food-insecure at least sometime during the year 2020 [2]. Additionally, 3.9% of U.S. households experienced severe food insecurity, characterized by decreased food intake and altered eating patterns among some family members due to resource scarcity. Previous literature from Peru, following a history of natural disasters, suggested a higher prevalence of food insecurity, which may resemble the long-term effects of other crises, such as COVID-19 [3, 4]. Food-insecure patients frequently struggle with episodic food shortages and a lower intake of nutritious foods, both of which are associated with the development of chronic illnesses [5].

Food insecurity and the incidence of chronic illness are linked, with a higher likelihood of poor chronic illness management due to difficulties in acquiring medications or maintaining consistent medical care [6, 7]. In 2010, data from the National Health and Nutrition Examination Survey (NHANES) of low-income participants showed that those reporting food insecurity were more likely to report hypertension, hyperlipidemia, and type 2 diabetes mellitus (T2DM) [8, 9]. Correlation studies also demonstrated a higher risk of cardiovascular disease and elevated A1c levels in households experiencing food insecurity [10, 11]. Furthermore, when comparing food-secure and food-insecure patients, the latter group was more likely to be low-income, minorities, smokers, and obese [12]. These factors, coupled with the higher prevalence of chronic diseases among food-insecure individuals, increase their risk of dying from cardiovascular disease [13]. However, the association between hyperlipidemia and food insecurity has shown inconsistent results; some studies have indicated moderate food insecurity is associated with dyslipidemia markers such as low high-density lipoprotein (HDL) cholesterol and high low-density lipoprotein (LDL) cholesterol levels in women, while others have found no relationship between the two factors [14-16].

Although food insecurity rates have declined in recent years, the rate in Alachua County remains at 13.4%, higher than the state average of 12%, according to data from the Florida Department of Health [17]. Alachua County is the location of the clinic utilized in this study. These data suggest a considerable population of food-insecure patients seeking primary care in the Gainesville area. To date, no known studies have examined the impact of food insecurity on chronic disease among residents of the Gainesville area. Therefore, determining the relationship between food insecurity and the prevalence and management of chronic disease in this population has clinical relevance for improving the prevention and control of chronic illnesses.

In this retrospective study of patients seeking medical care at UF Health’s Springhill Internal Medicine clinic, we investigated the relationship between three self-reported social determinants of health (food insecurity, physical activity level, and financial stability) and the prevalence and management of chronic diseases (T2DM, hypertension, and hyperlipidemia). We hypothesized that those experiencing food insecurity would have a higher prevalence of chronic illness (hyperlipidemia, T2DM, or hypertension) than those without food insecurity. Additionally, among those with a chronic disease, we hypothesized that food-insecure individuals would have poorer control of the disease than those who are not food-insecure, as determined by objective lab values. We also evaluated other factors, such as physical activity and financial stability, to assess potential confounding factors and explore the need for further research on these topics. Specifically, determining the relationship between food insecurity and the prevalence and management of chronic disease among residents of the Gainesville area may have clinical relevance as we work to prevent the development of chronic illness.

## Materials and methods

This retrospective chart review was approved by the Institutional Review Board at the University of Florida (UF), Gainesville, Florida.

Exclusion criteria included patients under 18 years of age, those over 85 years, those who declined to answer one or both of the screening questions (see Appendix 1), and those who answered the screening questions but did not have labs within one year of their visit. Inclusion criteria included all patients aged 18-85 years seen at UF Health Springhill Internal Medicine Clinic by a co-author from January through July 2022 who also answered the two standard screening questions (see Appendix 1) and received labs within one year of their visit. The screening questions are the standard questions given to all UF Springhill patients at check-in during their appointments. If patients denied both questions, they were labeled as food secure. If patients responded "yes" to either question, they were labeled as food insecure. The patient’s food insecurity status was then compared to their history of hypertension, T2DM, and hyperlipidemia. The management of each patient’s chronic illnesses was assessed using laboratory studies performed within one year of the appointment date, which were representative of overall control (see Appendix 2). In addition to food insecurity, other social determinants were recorded, including physical activity and financial stability. For physical activity, patients were labeled as sufficiently or insufficiently active based on the goal of exercising for at least 30 minutes on at least five days a week (see Appendix 1). For financial stability, the same method used for food insecurity was applied to label patients as financially stable or financially unstable (see Appendix 1). Demographics, including preferred language, age, gender, and BMI, were also collected from each patient’s EPIC (electronic patient information chart).

Each patient was assigned a unique code based on how their data were collected to avoid violating patient privacy (see Appendix 3). 

For statistical analysis, demographic and clinical variables were compared between the food-secure and food-insecure groups using the Wilcoxon Rank Sum and chi-squared tests. The power analysis was based on the primary analysis using the dichotomized food security measure. We assumed that the prevalence of one or more chronic diseases in the food-secure group would be half that in the food-insecure group (i.e., 33% vs. 16.5%, respectively). Additionally, we assumed that the prevalence of food insecurity would be 27%, based on prior data from the National Health and Nutrition Examination Surveys [6]. We set our target type 1 error rate at 0.05 and our target power at 0.8. With these parameters, our final study population of 349 provides sufficient power to detect the effects of food insecurity on chronic illness prevalence and management.

## Results

A total of 349 patients seen at the University of Florida Springhill Clinic by one co-author between January and July 2022 met the inclusion criteria for this study. The study population was predominantly female (71.35%, n=249) with a median age of 53 years, a median BMI of 29.61 kg/m², and English as the primary language (see Table 1).

**Table 1:** Demographics of the Population

Demographic	Category	Data
Gender	Female	71.35% (249)
	Male	27.79% (97)
	Did not report gender	0.86% (3)
Age median	53 years old (interquartile range 40-65)	
Average body mass index (kg/m^2^)	29.61 ± 6.61	
Primary language	English	70.49% (246)
	Spanish	29.23% (102)
	Other	0.29% (1)

**Figure 1:**
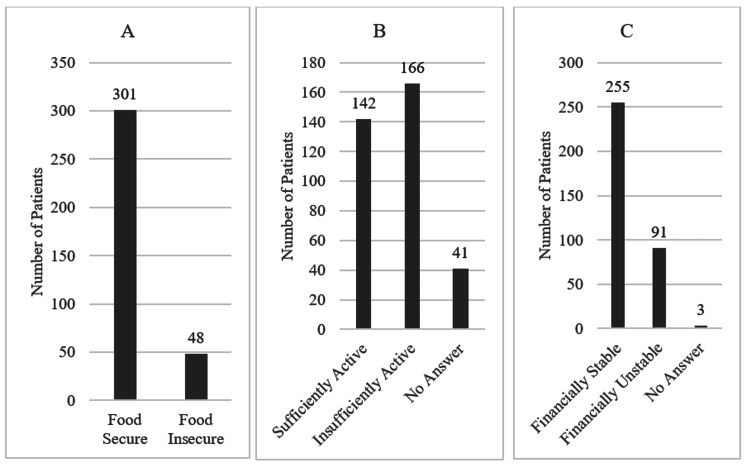
Self-Reported Social Determinants of Health (A) Prevalence of food insecurity. (B) Self-reported physical activity level. (C) Financial stability.

All patients included in the study answered the food insecurity questions; however, some did not respond to the remaining questions about other social determinants of health (see Figure 1). The totals across each category are as follows: 301 were food-secure, 48 were food-insecure, 142 were sufficiently active, 166 were insufficiently active (53.89%), and 41 did not answer the physical activity question. Additionally, 255 were financially stable, 91 were financially unstable (26.3%), and 3 did not answer the financial stability question. The overall prevalence of food insecurity in the study population was 13.75% (n=48).

**Figure 2:**
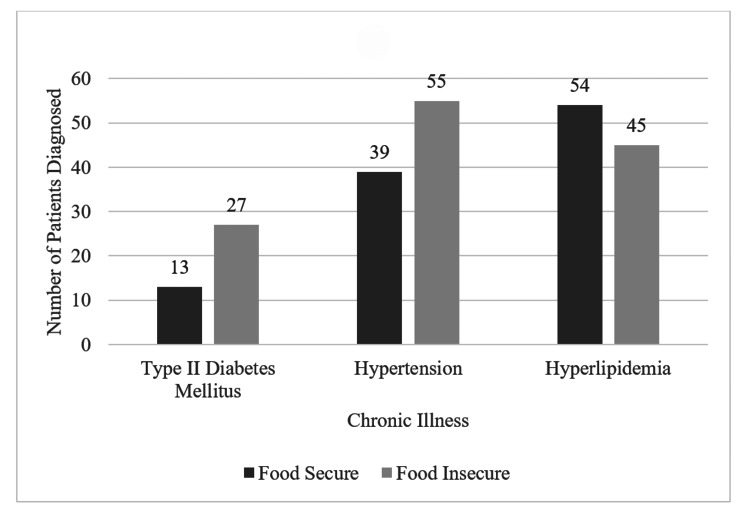
Association Between Food Insecurity and Prevalence of Chronic Illness

This study specifically investigated the connection between chronic illness diagnoses and whether the patient self-identified as food insecure (see Figure 2). There was an association between patients reporting worry about food running out and a diagnosis of hypertension (p = 0.019) and T2DM (p = 0.005). Similarly, there was also an association between patients reporting actually running out of food and a diagnosis of T2DM (p = 0.001).

**Figure 3:**
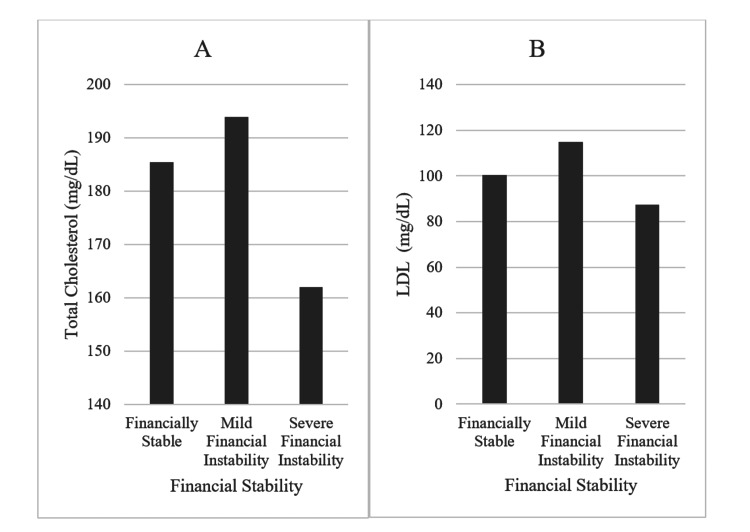
Associations Between Financial Stability and Chronic Illness Management Associations between (A) financial stability and total cholesterol (mg/dL) and (B) low-density lipoprotein (LDL) (mg/dL) levels.

Across the board, the lab values representing the quality of chronic illness management were not significantly different between the food-secure and food-insecure groups (see Figure 3). The exception to this observation includes the difference between the patients who reported never running out of food (food secure) and sometimes running out of food (food insecure); these two groups saw a difference in total cholesterol (p = 0.0415) and diastolic blood pressure (p = 0.0204). These values were slightly elevated in the food-insecure group.

Figure 3 shows how financial stability affects total cholesterol and LDL results. This is one objective measure of hyperlipidemia control, and it varied between different degrees of financial stability. The following are the corresponding p values per Wilcoxon Rank Sum regarding the difference in total cholesterol and financial stability: stable vs mild instability (p = 0.065), stable vs. severe instability (p = 0.047), and mild instability vs. severe instability (p = 0.0100). These are the values regarding the difference in LDL cholesterol and financial stability: stable vs. mild instability (p = 0.032), stable vs. severe instability (p = 0.166), and mild instability vs. severe instability (p = 0.024). 

## Discussion

This study aimed to identify the relationship between three self-reported social determinants of health and the prevalence and management of chronic diseases in the Gainesville area. First, we quantified the prevalence of food insecurity in this patient population, which was found to be 13.75% (n=48), remarkably similar to the average of 13.4% reported by Alachua County in 2019 (see Table 1). Second, we studied the association of diagnosed chronic diseases with the multiple responses given in a predetermined food insecurity questionnaire. We found an association with a coexisting diagnosis of T2DM regardless of whether the patient was only worried about food running out (p = 0.005) or did run out of food (p = 0.001) (see Figure 2). Additionally, we found an increased risk of hypertension (p = 0.019) and higher diastolic blood pressure (p = 0.0204) in this population.

Despite the discovered associations between food insecurity, T2DM, and hypertension, it is important to note that these factors could impact the health of each patient to varying degrees of severity and over vastly different time periods. This further emphasizes the need for screening, as it is unknown when a patient will be at the highest risk for morbidity or mortality. Inconsistent access to food suggests that this patient population would likely struggle to manage a diabetic-friendly diet and may have limited ability to manage their blood sugar. Still, the values for HbA1c were not statistically significant based on food security. Future studies may include other complications of uncontrolled T2DM, such as nephropathy, neuropathy, or retinopathy. Patients facing food insecurity also had an increased risk of hypertension (p = 0.019).

The other social determinants of health reviewed included physical activity and financial stability. Sufficient physical activity correlated with a decreased risk of chronic illness and resulted in more regular lab values as anticipated. While less statistically significant, financial stability had more unexpected associations with LDL and total cholesterol (see Figure 3). Overall, mild financial instability seemed to increase these values and suggest more severe hyperlipidemia, but the opposite was true for severe financial instability. There is no clear trend for these values, which disagrees with previous literature suggesting that higher socioeconomic status is associated with more severe dyslipidemia [18]. This portion of the study was inconclusive and may simply represent a population anomaly, requiring further investigation.

There are some limitations in our study that must be considered. First, we used data collected from a self-reported verbal questionnaire integrated into the electronic medical record at the University of Florida. This method potentially left patients vulnerable to embarrassment and likely reduced the number of patients reporting food insecurity, financial instability, or a lack of exercise. Furthermore, this questionnaire groups the patients by oversimplifying different factors, not taking into account that these social determinants of health exist on a spectrum based on each patient’s circumstances. Nonetheless, this allowed us to gather a larger sample size, and this study’s protocol could be easily replicated in additional clinics to offer further data and allow for conclusions that could be more widely applied. Another limitation is our small sample size (n=349), which limits the external validity of this study. Future studies should evaluate data from a larger cohort of patients. This questionnaire is also not a validated tool for assessing food insecurity, given that this study collected data from the charts and did not administer a survey specifically for the study. Future studies are encouraged to use validated food insecurity questionnaires. Another consideration is that recent studies have examined whether chronic disease predicts food burden [19], rather than if food burden predicts chronic disease. Further studies should attempt to explore these connections. Additionally, it must be noted that our study sample mostly consisted of females with a median age of 53 years, suggesting that the results are most pertinent to that population (see Table 1).

This study substantiates existing literature in a call for further screening of social determinants of health and raises questions about what can be done to mitigate the potentially elevated risk factors associated with the various outcomes. The long-term benefits of early screening and preventative measures to avoid chronic illness could be significant for both the patient and the overall cost of healthcare. While this study measured the prevalence of these risk factors and chronic illnesses, future research may seek not only to establish prevalence but also to enroll patients in appropriate prophylactic therapy.

## Conclusions

This study gathered data in accordance with the most recent published data on food insecurity prevalence by the Florida Department of Health in 2019, showing a food insecurity prevalence of approximately 13.4% in the Alachua County area. There was a statistically significant association between food insecurity and the diagnoses of type II diabetes mellitus and hypertension. No clinically applicable differences in the ability to manage chronic illness according to lab values were discovered, suggesting that patients would benefit most from primary prevention. Current questions exist regarding how successful brief screening surveys are for detecting clinically significant food insecurity and whether it would benefit the public to have more extensive screening. Regardless, there are clear risk factors associated with food insecurity that can affect clinical decision-making, and earlier identification could improve patient outcomes.

## Appendices

Appendix 1

Social Determinants of Health Screening Questions

Food insecurity: Patients were asked to comment on two statements regarding food insecurity on their intake forms: “Within the past 12 months, you worried that your food would run out before you got the money to buy more,” and “Within the past 12 months, the food you bought didn’t last, and you didn’t have money to get more.” They could respond with “never true,” “sometimes true,” or “often true.” If the patient answered “sometimes true” or “often true” to either question, the patient was labeled food insecure.

Physical activity levels: Then, the patients were grouped based on the self-reported quantity of exercise (Table 2).

**Table 2:** Self-Reported Physical Activity Levels and Assigned Group

Patient Self-Reported Quantity of Exercise	Assigned Group
The patient reports an amount of exercise equal to or greater than five days a week with 30 minutes per day	Sufficiently active
The patient reports an amount of exercise less than five days a week with 30 minutes per day	Insufficiently active

Financial stability: Patients were asked to comment on a statement on their intake forms regarding financial stability, choosing from “not hard at all,” “somewhat hard,” or “very hard.” The statement was: “How hard is it for you to pay for the very basics like food, housing, medical care, and heating?” Based on their responses, patients were grouped in two ways. The first, more broad categorization classified patients as financially stable if they answered “not hard at all.” Any response of “somewhat hard” or “very hard” placed the patient into the general group of financially unstable.

Appendix 2

To measure chronic illness management, laboratory study values were gathered and standardized values from the literature were used to label patients as controlled or uncontrolled (Table 3). 

**Table 3:** Standard Laboratory Study Values LDL: low-density lipoprotein.

Grouping patients according to standard laboratory values
1. Hypertension [20]
- Controlled: Blood pressure reading below 135 mmHg systolic and 85 mmHg diastolic for adults aged under 80 years old or below 145 mmHg and 85 mmHg diastolic for adults aged over 80 years old
- Uncontrolled: Blood pressure reading above or equal to 135 mmHg systolic and 85 mmHg diastolic for adults aged under 80 years old or above or equal to 145 mmHg systolic and 85 mm Hg diastolic for adults aged over 80 years old
2. Type 2 diabetes mellitus [21]
- Controlled: <7% hemoglobin A1c
- Uncontrolled: ≥7% hemoglobin A1c
3. Hyperlipidemia [22]
- Controlled: Fasting LDL less than 160 mg/dL
- Uncontrolled: Fasting LDL greater than or equal to 160 mg/dL

Appendix 3

All data were entered into the REDCap data capture software without any patient identifiers. To maintain data privacy, each patient was assigned a unique code based on their appointment date and the data collector assigned to them. This code included a number representing the order in which the patient was seen relative to others on the same day, the day they were seen, and the data collector’s initials. Initially, all patient data, including those who did not meet the inclusion criteria, were input into the program. Once the data were collected and verified by the research team, both the identifying codes and the data of patients who failed to meet the inclusion criteria were removed.
